# A Practical EEG-Based Human-Machine Interface to Online Control an Upper-Limb Assist Robot

**DOI:** 10.3389/fnbot.2020.00032

**Published:** 2020-07-10

**Authors:** Yonghao Song, Siqi Cai, Lie Yang, Guofeng Li, Weifeng Wu, Longhan Xie

**Affiliations:** Shien-Ming Wu School of Intelligent Engineering, South China University of Technology, Guangzhou, China

**Keywords:** EEG, human-machine interface, assist robot, online control, practicability

## Abstract

**Background and Objective:** Electroencephalography (EEG) can be used to control machines with human intention, especially for paralyzed people in rehabilitation exercises or daily activities. Some effort was put into this but still not enough for online use. To improve the practicality, this study aims to propose an efficient control method based on P300, a special EEG component. Moreover, we have developed an upper-limb assist robot system with the method for verification and hope to really help paralyzed people.

**Methods:** We chose P300, which is highly available and easily accepted to obtain the user's intention. Preprocessing and spatial enhancement were firstly implemented on raw EEG data. Then, three approaches– linear discriminant analysis, support vector machine, and multilayer perceptron –were compared in detail to accomplish an efficient P300 detector, whose output was employed as a command to control the assist robot.

**Results:** The method we proposed achieved an accuracy of 94.43% in the offline test with the data from eight participants. It showed sufficient reliability and robustness with an accuracy of 80.83% and an information transfer rate of 15.42 in the online test. Furthermore, the extended test showed remarkable generalizability of this method that can be used in more complex application scenarios.

**Conclusion:** From the results, we can see that the proposed method has great potential for helping paralyzed people easily control an assist robot to do numbers of things.

## Introduction

Electroencephalography (EEG), a meaningful attempt to explore the secrets of the brain which is made up of neurons (Noctor et al., 2001). It also can be used to reflect the human intention under different physiological conditions because the transfer of information between neurons produces changes in electrical potentials (Schomer and da Silva, 2012). High-resolution EEG (Mitzdorf, 1985) is quickly becoming a powerful tool in human-machine interface (HMI), with which people is able to work by internal intention and external equipment instead of their own limbs (Gao et al., 2003). This technology plays a vital role in helping disabled people out of the dilemma that they have to rely on the help of others all the time. Moreover, robots can be controlled by EEG-based HMI to assist paralyzed people with neuromuscular disorders such as stroke or amyotrophic lateral sclerosis in performing rehabilitation training (Prasad et al., 2010; Ang et al., 2015). A large amount of evidence shows that EEG-based assist robots effectively promote patients recover (Bhattacharyya et al., 2014; Chaudhary et al., 2016; Alia et al., 2017; Monge-Pereira et al., 2017; Cervera et al., 2018) by essentially helping reconstruct the neural circuit between the brain and the muscles compared to the traditional method of repetitive motion (Dobkin, 2004; Belagaje, 2017).

Many control methods for assist robots have emerged but there are still some challenges such as causing fatigue, requiring too much pre-training, or poor online testing results (Tariq et al., 2018), which allows us to question how assist robots can be more practical for assistance purpose. Steady-state visually evoked potentials (SSVEPs) are adopted to extract effective components of EEG with too much, and cluttered information. An SSVEP-controlled assist robot has been developed to perform pick and place tasks (Chen et al., 2019), which are completed by showing the user four targets flickering at 30, 31, 32, 33 Hz to trigger different commands. In the same regard, SSVEPs are used to control functional electrical stimulation for rehabilitation (Han-Pang, 2015). This paradigm has good information transfer rate (ITR) and accuracy (Vialatte et al., 2010; Chen et al., 2019), but is restricted by the limited number of control commands (Norcia et al., 2015; Zhao et al., 2015), and constant flickering easily causes fatigue, which is not appropriate for people in rehabilitation (Duszyk et al., 2014). Some research turns its attention to motor imagery (MI) for closely correlating brain commands and body movement responses. A system with MI for after-stroke rehabilitation exercises has been presented (Wang et al., 2009), that controls a robot to drive the arm by letting subjects imagine hands moving. In another work, the left, right, up, and down robot arm movements are driven by imagining the movement of their left hand, right hand, both hands, and the relaxation of both hands (Meng et al., 2016). Nevertheless, MI is not practical enough because the imaginary movements with distinct individual differences are not definite, causing participants to need extra pre-training and resulting in lower accuracy (Dahm and Rieger, 2016).

Event-related potentials (ERPs), brain voltage fluctuations in response to specific stimuli such as images or sounds (Sams et al., 1985; Picton et al., 2000), provide a more straightforward mean to control the assist robot. A lower limb prosthesis based on P300, the peak observed 300 ms (250–500 ms) after a specific event (Picton, 1992; Polich, 2007), has been developed to help people walk (Duvinage et al., 2012). Four letters for simulation are used to represent low-, medium-, and high-speed states and stop states. As one of the most easily observed ERP, P300 is also used for spelling by constantly flashing the rows and columns of a 6 × 6 alphabet matrix when the subjects are focusing on a target letter, which stimulates the P300 response (Velasco-Álvarez et al., 2019). Therefore, we chose P300 because it was more stable to be observed in most people and less prone to fatigue.

Researchers have tried some methods to detect P300 (Raksha et al., 2018; Tal and Friedman, 2019; de Arancibia et al., 2020) and no good conclusion yet as to which method is really acceptable to practical online use of assist robot due to the rigorous requirement for accuracy and stability. Many good works with impressive performance have been obtained (Cecotti and Graser, 2011) only from offline experiments (Kobayashi and Sato, 2017; Mao et al., 2019; Kundu and Ari, 2020) or BCI competition (Kundu and Ari, 2018; Arican and Polat, 2019; Ramele et al., 2019). However, we cannot risk using offline results directly in real life. Serious differences may be caused by complex objective factors such as impedance (Ferree et al., 2001) and specificity (Chowdhury et al., 2015). Some researchers have also tried online experiments, but many of them focus on new frameworks or user interfaces while ignoring the performance of the method itself (Achanccaray et al., 2019; Lu et al., 2019; Mao et al., 2019).

Consider these issues, we propose a highly practical control system with remarkable accuracy and nice ITR after comparing three P300 detection methods offline and online, that can be used for the control of assist robots. Moreover, with the method, a complete upper-limb assist robot has been built to verify the feasibility and effectiveness of the system, as well as an exploratory mobile robot controlled together with computer vision to confirm the robustness and generalizability.

The main contributions of this research are as follows: (i) a handy and efficient EEG-based method to steadily control assist robots for helping disabled people practically was proposed; (ii) With the method, we developed a safe robot that can assist user to perform up to 36 preset upper-limb movements such as rehabilitation exercises; (iii) both offline and online tests were performed to verify the practicability of the finalized system with detailed comparisons of three classifiers as intention detector; and (iv) good reference for future research was provided by a novel assistive mobile robot with shared control of our method and computer vision.

The sections of this paper are given as follows. We introduce data collection, the framework of our control method, the theoretical details of three comparison methods, the composition of the upper-limb assist robot and the extended test in section Materials And Methods. The experiments and the results are presented in section Results. Finally, the study is discussed in section Discussion, and concluded in section Conclusions.

## Materials and Methods

### Data Acquisition

Eight healthy volunteers (six males and two females), aged from 19 to 28 years old, joined in these experiments. All participants without any experience of an EEG experiment gave informed consent before the experiment. All the procedures were approved by the Guangzhou First People's Hospital Department of Ethics Committee. It took <2 h for each one to complete the experiment. EEG signals recorded from 64 scalp electrodes on BrainCap MR (Brain Products GmbH) were amplified using BrainAmp MR (Brain Products GmbH), with all impedances kept at approximately 20 kΩ.

The P300 speller paradigm (Farwell and Donchin, 1988) composed of 26 letters and 10 numbers in a 6 × 6 matrix was used for stimulation. Each participant was instructed to watch a 15.6" LCD monitor 0.7 m away with a 6 × 6 character matrix and focus on one of them. The character flashed within the entire row or column, and each row or column flashed once for a total of 12 times per repetition. Twelve repetitions were conducted to recognize one character. In the training portion of the experiment, the rows and columns flashed in random order with a flashing duration of 100 ms and a no-flash duration of 50 ms, leading to an interstimulus interval (ISI) of 150 ms. Besides, we set the inter-repetition delay to 0.5 s and the inter-character delay to 5 s. Fifteen characters were collected for training classifiers, which took 476.5 s for each participant to perform. A dataset for specific people and objective factors was obtained with 360 target samples and 1,800 no-target samples, comprising 2,160 samples in total.

### Method

**Figure 1:**
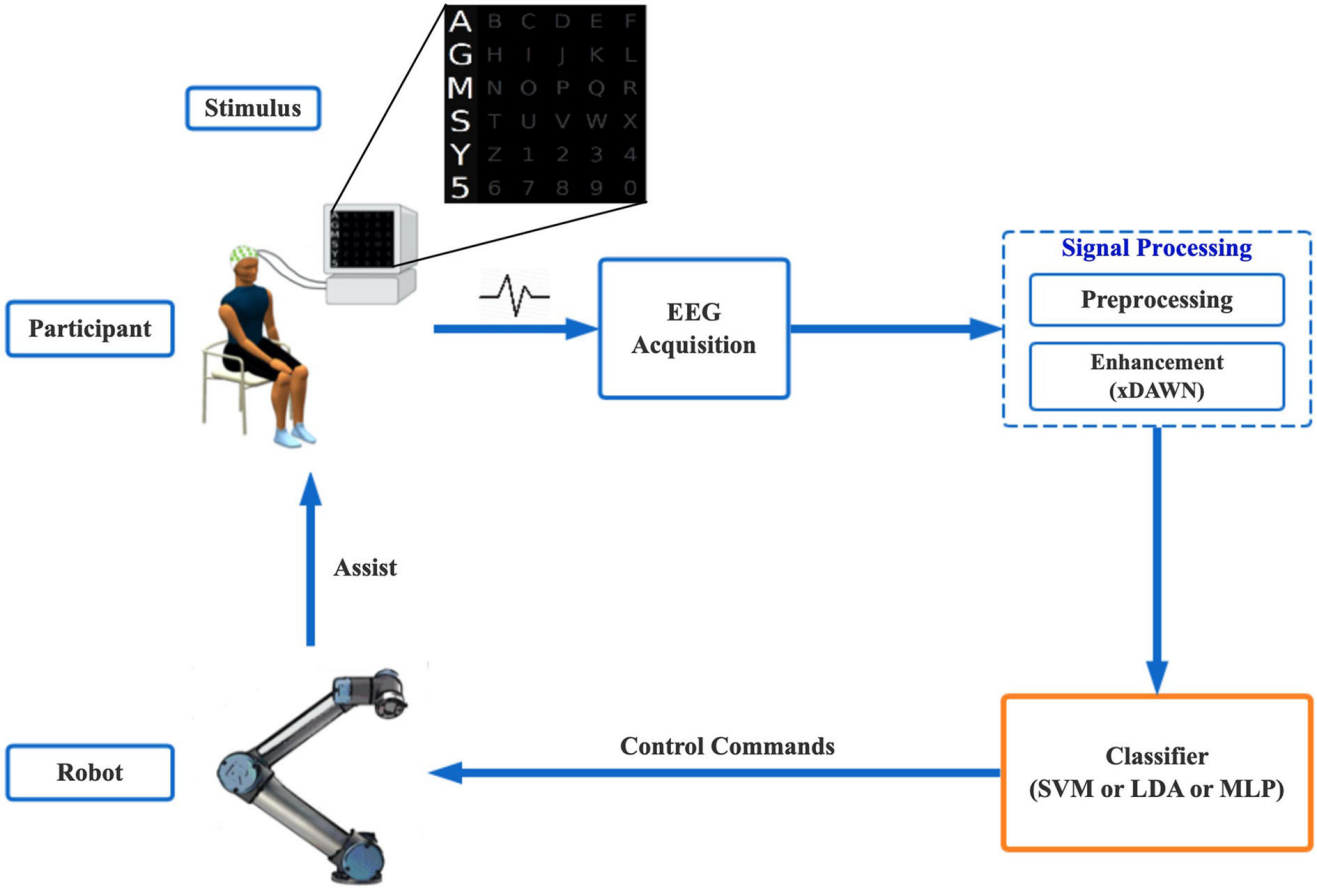
Schematic diagram of the proposed EEG-based assist robot control method.

The schematic diagram of the proposed method is shown in Figure 1. It consists of four parts: the user module to stimulate participant and acquire EEG signal, the signal processing part to conduct pre-processing and feature enhancement, the classifier to detect P300 from continuous EEG, and the execution module to control the robot. In the short-term training phase, the acquired data were pre-processed, and a spatial filter was used to enhance the features. Then, we used the processed data to train the P300 classifier. In the online test phase, the data were classified to obtain the user's intention as control commands for the assist robot.

### Pre-processing and Data Enhancement

First, the signals collected at a sampling rate of 500 Hz were temporally filtered using a fourth-order Butterworth bandpass filter with a 1 Hz lower cut-off frequency and a 20 Hz upper cut-off frequency. Then, down sampling with a factor of four was used to reduce the computational cost. Next, the signal was divided into 500 ms epochs, including 250 ms duration to preserve the P300 wave and a 250 ms interval to avoid overlap.

After the above steps, the spatial filter based on xDAWN algorithm (Rivet et al., 2009) was used to enhance the evoked potential of the data which means to improve the signal to signal-plus-noise ratio (SSNR). It can be expressed as follows:

(1) $$\begin{array}{r} EEG_{pp}=D_{1}A_{1}+D_{2}A_{2}+N \end{array}$$

where *EEG*_*pp*_ is the matrix of preprocessed EEG signal. *A*_1_ and *A*_2_ indicate two states with evoked potential and no evoked potential. *D*_1_ and *D*_2_ are Toeplitz matrices whose first column are defined to zero except for those corresponding to the two states. *N* is the sum of the original noise. *A*_1_ can be factorized as $a_{1}W_{1}^{T}$, where *a*_1_ is the temporal distribution and *W*_1_ is the spatial distribution. Now the SSNR can be defined as:

(2) $$\begin{array}{r} SSNR=\frac{U^{T}E_{1}U}{U^{T}E_{E}U} \end{array}$$

where *U* is a spatial filter, *E*_1_ and *E*_*E*_ represent the expectation of ($A_{1}^{T}D_{1}^{T}D_{1}A_{1})$ and ($EEG_{pp}^{T}EEG_{pp})$ separately. Assuming that *A*_1_ is uncorrelated to other parts, the *SSNR* can be rewritten as:

(3) $$\begin{array}{r} SSNR=\frac{{E_{1}}^{\prime }{\left(U^{T}W_{1}\right)}^{2}}{U^{T}({E_{1}}^{\prime }W_{1}W_{1}^{T}+D_{2}A_{2}+N)U} \end{array}$$

where ${E_{1}}^{\prime }$ is the expectation of ($a_{1}^{T}D_{1}^{T}D_{1}a_{1})$. The spatial filter can be calculated by maximizing *SSNR*. The *A*_1_ can be obtained by least mean square estimation, then the estimated spatial filter Û. The final output after pre-processing and spatial enhancement can be calculated by:

(4) $$\begin{array}{r} EEG_{p}=EEG_{pp}\;Û \end{array}$$

where *EEG*_*p*_ is the matrix of processed EEG signal. The signal space is compressed to three dimensions, which serves as an input to make the classifiers of the next part substantially more efficient.

Due to the good performance of xDAWN, channel selection was no longer a problem that seriously affected the experiments. All 64 channels were used to provide sufficient information without worrying about too much computation for the classifier. From another perspective, we can settle for impedances at approximately 20 kΩ to cut down the preparation time before the experiment.

### Classifiers

The EEG with complex structure is easily affected by slight changes in human thoughts. Therefore, a highly efficient and robust classifier is essential for P300 detection. Some classification methods have been used for P300 detection, such as linear discriminant analysis (LDA), random forest (RF), and convolutional neural network (Cecotti and Graser, 2011; Duvinage et al., 2012; Akram et al., 2015; Hong and Khan, 2017). Some good results have been achieved with some datasets but testing using real-time data is insufficient. As we all know, there are many external factors that affect the signal quality of non-invasive EEG. It makes us confused that what is most suitable for assist robot online control. Besides, too much time is unacceptable to train a large amount of data with a complex classifier in rehabilitation scenario. Thus, the classifier design is a serious problem in our work.

To propose a method capable of accurately detecting the P300 signal at a fast speed, we used three types of classifiers– SVM, LDA, and multilayer perceptron (MLP) –to classify the data into two classes, Target meant P300 signal was detected and No Target meant no P300 signal was detected.

For every participant, only the data of 15 characters were used as the training set, which was acquired as described in section Data acquisition. Five-fold cross-validation was introduced to avoid overfitting. The following content will explain the structures and parameter settings of the three classifiers in detail.

#### LDA Classifier

Linear discriminant analysis is a classification algorithm which performs well for EEG signals (Xu et al., 2019) despite its simple structure. It looks for a vector to preserve as much information indicating the discrimination of class Target and No Target as possible, which means to make the samples in the same class more aggregated and those from different classes more separated. LDA was used to verify whether the cost-effective linear classifier could be used well for EEG signals. The low input dimensions made the dimensionality reduction mapping smoother as well. For better classification results, a metric function was used as:

(5) $$\begin{array}{r} J\left(V_{EEG}\right)=\frac{V_{EEG}^{T}S_{b}V_{EEG}}{V_{EEG}^{T}S_{w}V_{EEG}} \end{array}$$

where *V*_*EEG*_ is the vector used to project the output from EEG signal processing part to a low-dimensional sample space for discrimination; *S*_*b*_ is the between-class scatter representing the distance between the means of the classes; and *S*_*w*_ is the within-class scatter, which is the variance within a class. These two scatters were used to control Target away from No Target and to ensure that each class was dense enough. The partial derivative given as follows was used to find *V*_*EEG*_, which can obtain a maximum *J*(*V*_*EEG*_ ):

(6) $$\begin{array}{r} \frac{\partial J\left(V_{EEG}\right)}{\partial V_{EEG}}=S_{b}V_{EEG}-J\left(V_{EEG}\right){\;S}_{w}V_{EEG} \end{array}$$

Through the determinant operation, we can obtain the values of *J*(*V*_*EEG*_), further finding *V*_*EEG*_ which could be used for classification directly.

#### SVM Classifier

Support vector machine is a supervised machine learning method that is used for classification or regression. Similar to LDA, the processed EEG signal is mapped to high-dimensional feature space and is divided into two regions by a hyperplane. Considering the significant individual differences of different people's EEG signal, SVM, which does not require too many parameters or local optimization but has low generalization error, is particularly well-suited.

The training data is given as ${\left\{EEG_{i},L_{i}\right\}}_{i=1}^{n}$, in which *EEG*_*i*_ is the processed EEG signal segment and *L*_*i*_ ∈ {−1, 1} is the class label. We can determine the hyperplane by maximizing the separation margin of class Target and No Target and minimizing the classification error. It can be expressed mathematically as:

(7) $$\begin{array}{r} arg\;\max_{w,b}\left\{\frac{1}{\left||w|\right|}\;\min\left[L_{i}\left(w^{T}\varphi \left(EEG_{i}\right)+b\right)\right]\;\right\} \end{array}$$

where *w* is the weight and *b* is the bias, φ() is the mapping function. After modification we got:

(8) $$\begin{array}{r} \min\frac{1}{2}||w|{|}^{2}+C\overset{N}{\underset{i=1}{\sum }}\xi_{i}\;s.t.\;L_{i}\left(w^{T}\varphi \left(EEG_{i}\right)+b\right)\geq 1-\xi_{i} \end{array}$$

where ξ_*i*_ ≥ 0 is the slack variable representing the magnitude of the error. Further, the dual representation obtained with Lagrange multiplier method is:

$$\begin{array}{rcl} \text{max }L\left(a\right) & = & \overset{N}{\underset{i=1}{\sum }}a_{n}-\overset{N}{\underset{i=1}{\sum }}\overset{N}{\underset{j=1}{\sum }}a_{i}a_{j}L_{i}L_{j}\varphi {\left(EEG_{i}\right)}^{T} \end{array}$$

(9) $$\begin{array}{r} \varphi \left(EEG_{j}\right)\;s.t.\;\overset{N}{\underset{i=1}{\sum }}a_{n}L_{n}=0 \end{array}$$

where multiplier *a*_*n*_ ≥ 0. A non-linear kernel as follows was used in the classifier to replace the simple inner product of the data mapping which makes it more robust for the linearly inseparable case and differentiate from the previous linear method LDA. Then, the decision could be made with the hyperplane.

(10) $$\begin{array}{r} kernel=K\left(\varphi \left(EEG_{i}\right),\varphi \left(EEG_{j}\right)\right)\;={\left(\varphi {\left(EEG_{i}\right)}^{T}\varphi \left(EEG_{j}\right)+1\right)}^{3} \end{array}$$

#### MLP Classifier

**Figure 2:**
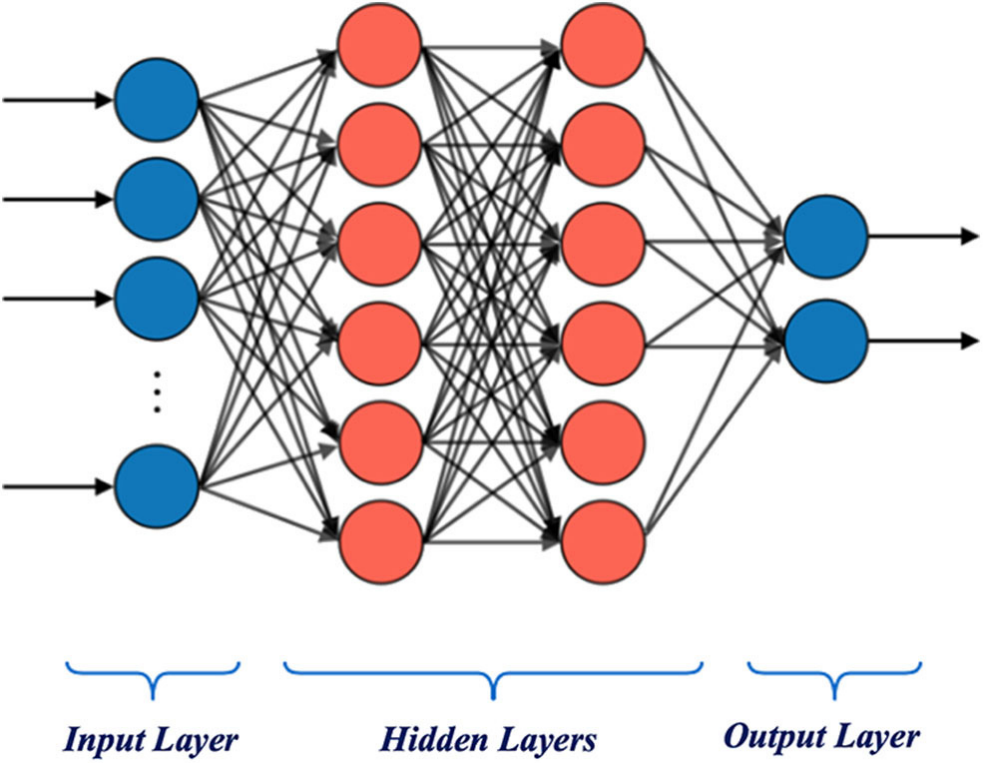
Structure of multilayer perceptron neural network.

Recently, deep learning based on neural networks has achieved amazing performance in some classification problems (Lecun et al., 2015). However, too deep network on small data is prone to problems like overfitting. MLP, a classic model with most characteristics of neural networks, was chosen for comparison. The structure of MLP shown in Figure 2 is made of three parts with layers containing many neurons that perform linear weighting calculation separately. Similar to the information step-by-step transfer in the brain, the processed EEG data input from the first layer is used to obtain few abstract features in the two hidden layers, and these features are integrated by the output layer to obtain the final classification result.

The tanh function given as follows was used as the activation function in the neurons to introduce non-linear properties after performing a linear operation on each neuron.

(11) $$\begin{array}{r} neuron\;ouput=tanh\left(F_{EEG}\right)=\frac{2}{1+e^{-2F_{EEG}}}-1 \end{array}$$

where *F*_*EEG*_ indicates the abstract feature of EEG in different layers. The weights and biases were corrected by gradient descent and calculated by backpropagation in training. With continuous optimization, this network was able to classify a sample to class Target or No Target at last.

### Upper-Limb Assist Robot System and Practical Test

**Figure 3:**
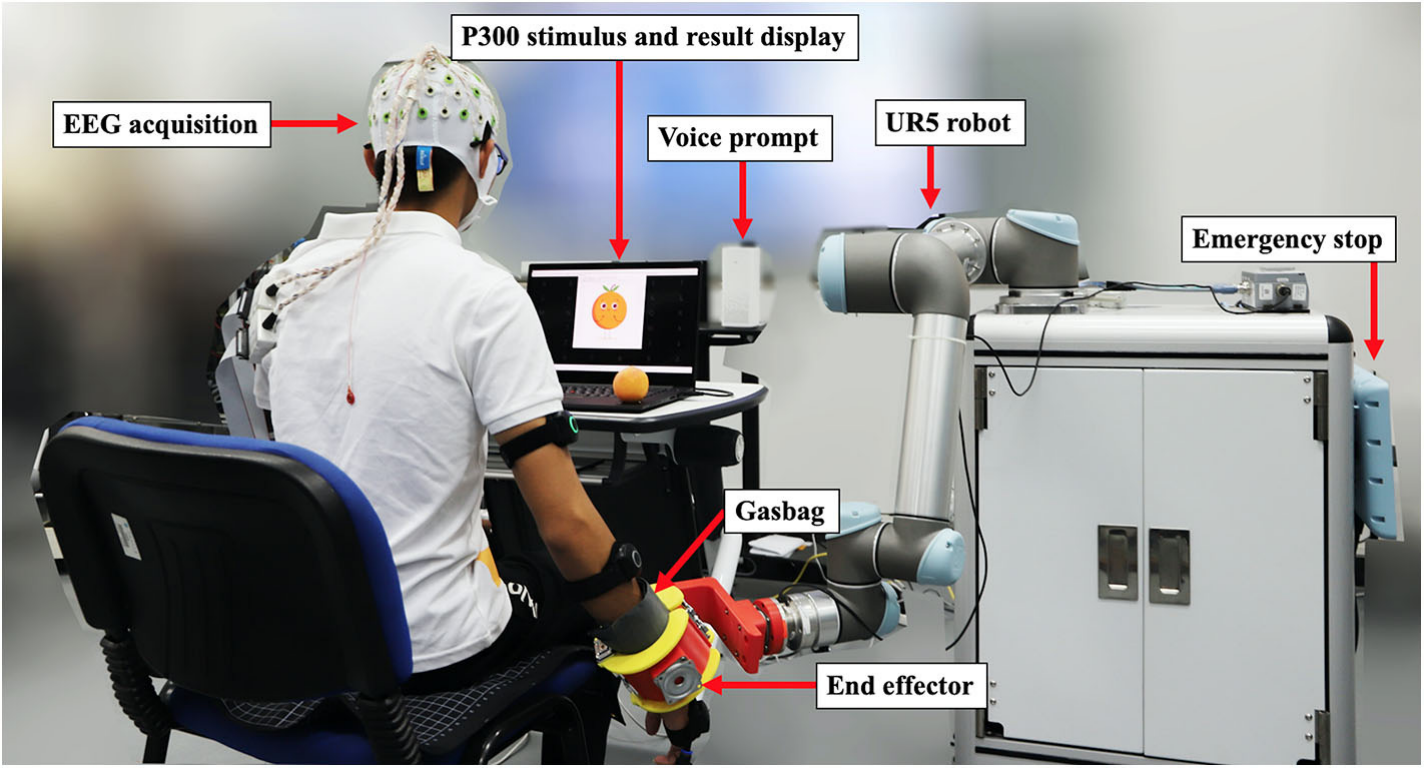
The upper-limb assist robot controlled by our method for testing. Written informed consent was obtained from the individual for the publication of this image.

We built an integrated control system to verify the actual performance of our methods and an upper-limb assist robot that can be used in practice, shown in Figure 3, which we hoping to employ to replace repeating mechanical rehabilitation that is commonly used in hospitals with general results but massive manpower. An example is given to illustrate the use, when the participant keeps focusing on the letter “O,” it would be detected by the system and accompany the screen displayed the corresponding indicative picture with a voice prompt to remind the user that the assistive movement of grasping orange was about to be done. After that, the robot helped the participant pick up the orange in front of him and put it down in another place. We could also program different actions for rehabilitation corresponding to the 36 characters. Besides, different quantities and forms of P300 stimulation, such as vivid pictures instead of characters, could be customized according to actual needs.

**Figure 4:**
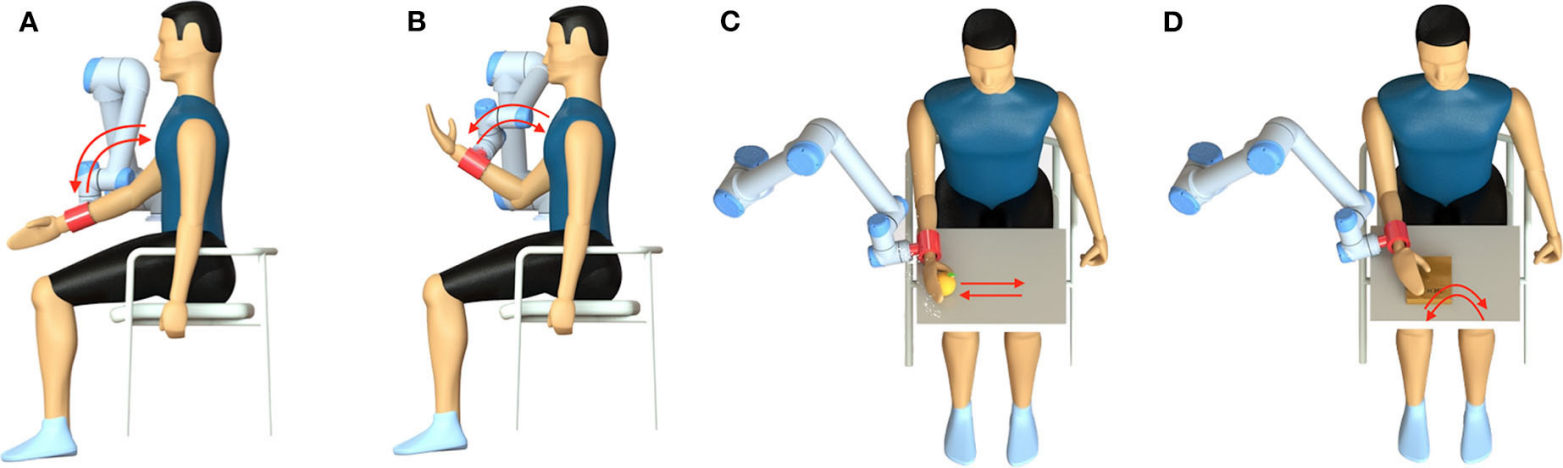
Four preset tasks of the upper-limb assist robot. **(A)** Shoulder flexion; **(B)** elbow flexion; **(C)** orange grasping; **(D)** book turning.

The control module was a laptop with an Intel Core i7-8750H processor and 16 GB RAM. The EEG signal was transmitted to this laptop after acquisition and amplification. We used OpenViBE (Renard et al., 2010) and MATLAB R2018b for data collection, signal processing, and user interface building. After detecting P300, the participant's intention is converted to a control command and sent to the execution module through the TCP protocol. The execution module was mainly composed of a UR5 robot (Universal Robots) for performing preset movements and a 3D printed end effector with a gasbag to assist the user in doing tasks by driving user's upper limb. As shown in Figure 4, we preset four tasks related to the shoulder and elbow muscle groups, shoulder flexion in Figure 4A, elbow flexion in Figure 4B, orange grasping in Figure 4C and book turning in Figure 4D, which were triggered separately by focusing on the corresponding character. Up to 36 movements that can be set give the assist robot a lot of possibilities.

We must pay attention to the security issue. The security mechanism of our assist robot included several aspects. There was an emergency stop button on the control panel with which the operator can stop the robot at any time. Besides, the force that the UR5 can withstand was limited to 80 N. If unexpected movement occurs, it will automatically stop without harming the user due to the small resistance generated by the user. Moreover, the preset movements would be recorded manually for each new user based on his exclusive status.

In practical use, it is not necessary to reduce the impedance to an excessively low level because of the good performance of our control strategy, preparation can be finished within a short time by an operator familiar with the process. According to the results of the pre-experiment, we reduced the ISI to 75 ms, consisting of a flashing duration of 50 ms and a no-flash duration of 25 ms, as well as an inter-repetition duration of 0.25 s. This measure was a substantially effective use of the P300 signal.

### Extended Test

We have built a control system with an upper-limb assist robot, and hope that the control strategy can be applied in a wild range of scenarios. Therefore, we develop a more novel mobile robot controlled by our HMI and computer vision for further verification.

**Figure 5:**
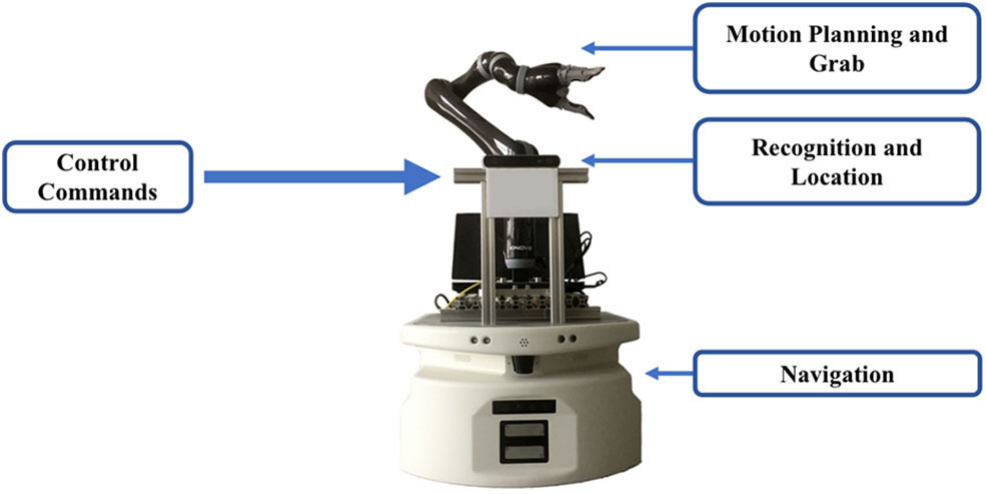
A novel mobile robot with control of our method and computer vison.

**Figure 6:**
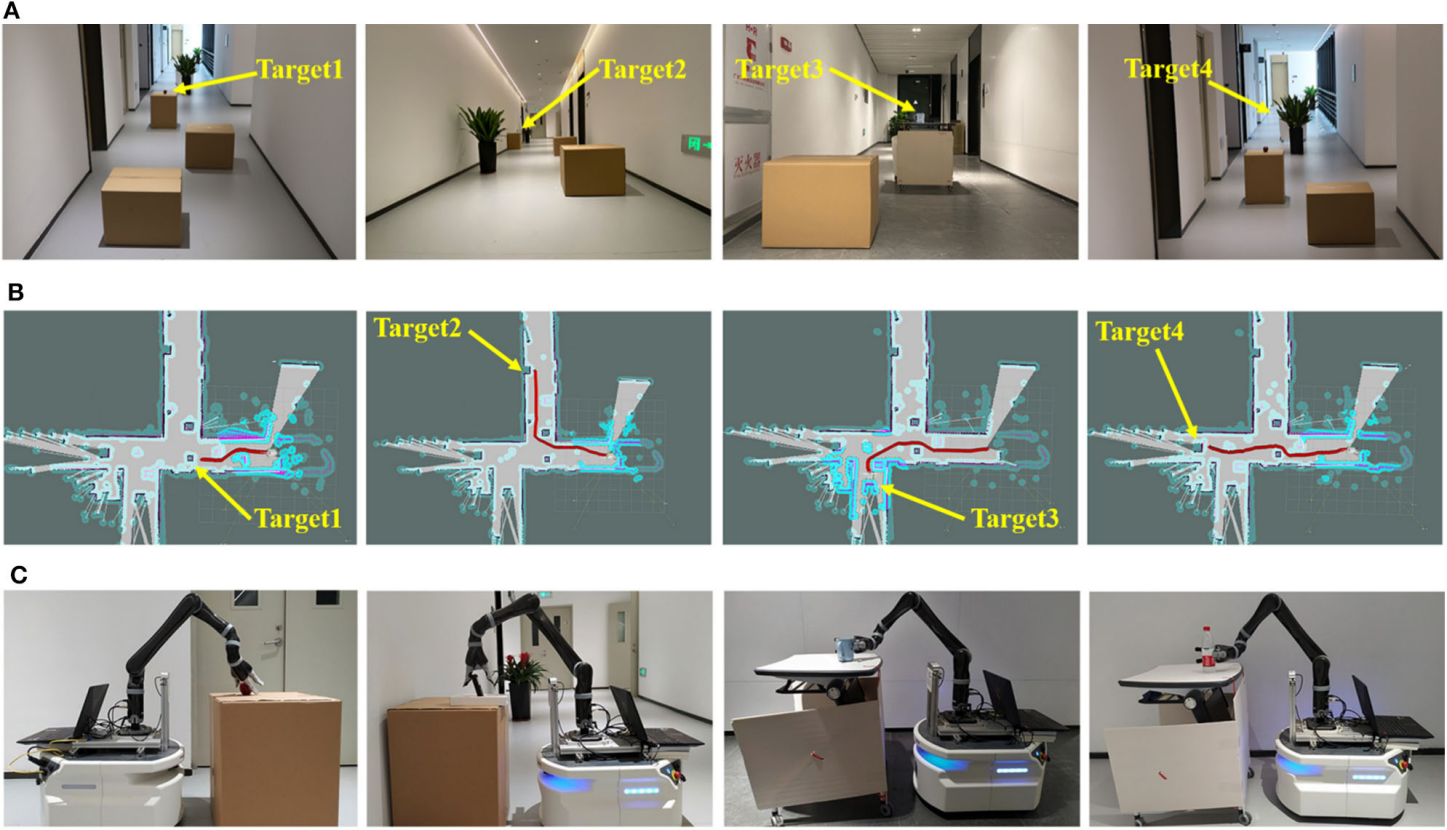
Scenarios of four targets for mobile robot testing. **(A)** actual scene; **(B)** route planning; **(C)** grasp motion.

As in the previous section, the control system was used to obtain the user's intention and control the mobile robot to go to another place to grasp items and return to the user. The mobile robot given in Figure 5 has three parts, a mobile platform with radar for navigation, a stereo camera (Stereolabs Inc.) for recognition and a robot arm (Kinova Inc.) for grasp. As shown in Figure 6A, we selected an area in which four positions had placed an apple, a mobile phone, a cup and a bottle as four targets to be grasped. Some cartons were randomly placed as obstacles.

We set four characters to correspond to the four targets and built a map of the environment at first in actual use. When the control system detected the participant's intention, the command would be wirelessly transmitted to the mobile robot. Then the mobile platform planned a global route to avoid the obstacles and reach the target with A^*^ algorithm as in Figure 6B. Near the target, the stereo camera used computer vision method (Redmon and Farhadi, 2017) to detect the target and calculate the three-dimensional coordinates, with which the robot arm could grasp the target as in Figure 6C. Finally, the mobile robot returns to the participant's position.

This part is an imperfect exploratory attempt. We hope to use it to further test the practicality and robustness of our control method, which showed a very high application value. There is no very detailed implementation description because the robot and computer vision technology we used is quite mature.

## Results

The 64-channel EEG data of eight healthy participants with no experience of P300 experiments were used to test our method. In each experiment, we collected data from 15 characters with a total of 2,160 samples for training and offline testing, followed by nine groups with 10 characters each for online testing. The linear LDA, non-linear SVM and neural network method MLP were compared with three experimental groups to obtain an effective method for the HMI.

**Figure 7:**
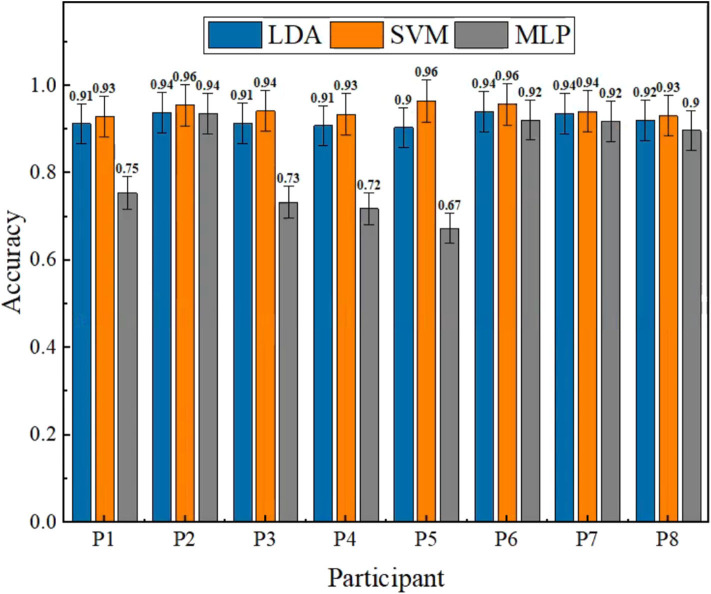
Offline results of three methods in eight different participants. The average accuracies with standard deviations of LDA, SVM, and MLP are 92.15 ± 1.42, 94.43 ± 1.32, and 81.87 ± 10.9%, separately. The results of MLP are significantly lower than LDA (*p* < 0.05) and SVM (*p* < 0.05).

The accuracy was used as the primary criterion to guarantee the reliability of the entire system after selecting the ISI and the number of repetitions. The training results of the three methods for each participant are presented in Figure 7 with average accuracies and standard deviations of 92.15 ± 1.42, 94.43 ± 1.32, and 81.87 ± 10.9%, respectively, in which we can see that LDA and SVM were always high and stable. The results of MLP were unstable and significantly lower than LDA (*p* < 0.05) and SVM (*p* < 0.05), even though it could achieve good accuracy in some cases.

Five-fold cross-validation was used to avoid overfitting and confirm the validity of our offline test because EEG with an amount of noise was easily affected by the limited data we used to reduce the detection ability of the method. The data of each participant was randomly divided into five equal parts. The five results obtained by using one of them as the test data and the other four as the training data were combined into the final accuracy. As shown in Table 1, the cross-validation accuracy of LDA and SVM remain stable with low standard deviation. However, MLP still shows large differences in different participants, sometimes having rather poor results. Besides, we used the standard deviation of the classification accuracy and cross-validation accuracy to evaluate the performance of three classifiers. The results of LDA, SVM and MLP are 0.0227, 0.0267, and 0.3200. It can be seen that the first two meet our demand, while the third is worse.

**Table 1:** Accuracy of 5-fold cross-validation.

**Participant**	**P1**	**P2**	**P3**	**P4**	**P5**	**P6**	**P7**	**P8**	**Average**
LDA	88.29	92.27	88.61	86.94	86.76	92.27	91.62	89.68	89.56 ± 2.27
SVM	88.84	93.70	90.37	89.07	86.90	92.22	92.50	90.97	90.57 ± 2.24
MLP	16.67	90.03	17.33	11.19	12.25	87.39	86.75	84.75	50.80 ± 39.02

*The standard deviations between these results and offline results of LDA, SVM, and MLP are 0.0227, 0.0267, and 0.3200, which shows that the offline performance of the first two meets our requirement, the third is worse*.

**Figure 8:**
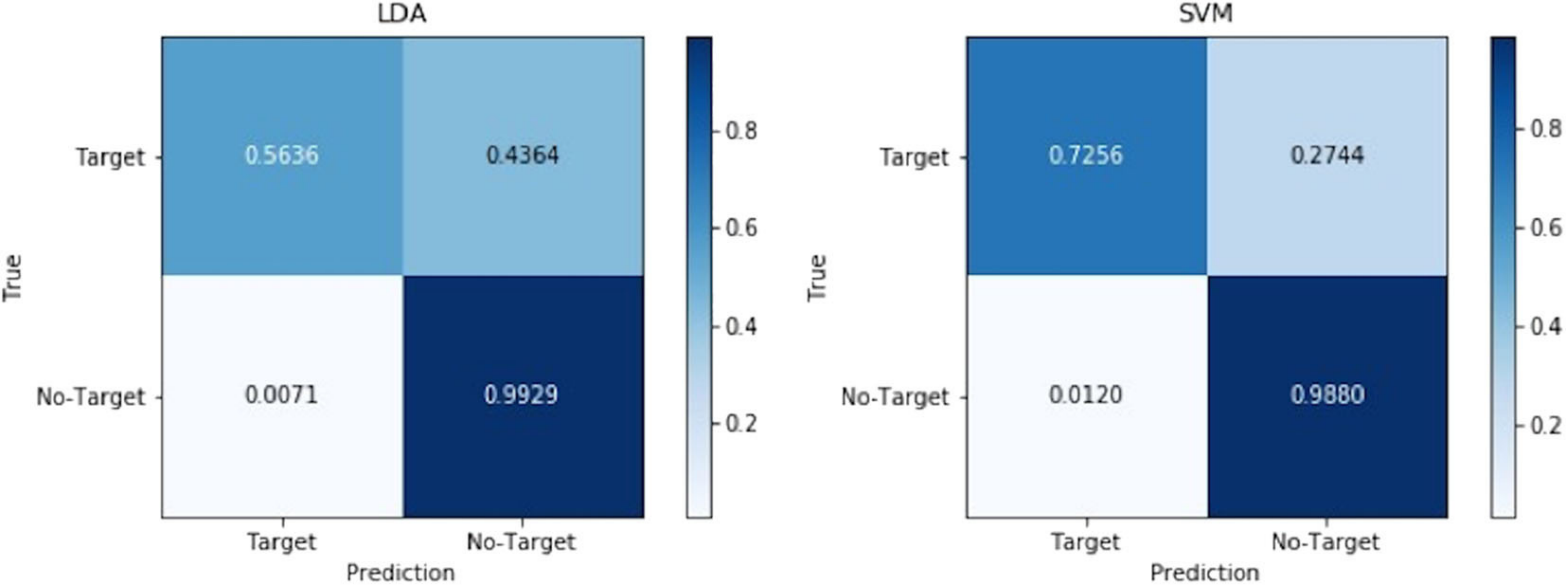
Normalized confusion matrices of LDA and SVM.

Then the confusion matrixes shown in Table 2 were used to compare LDA and SVM and further confirm the reliability of the finalized control method. Signals of target stimuli are referred to as positive samples, and signals of no-target stimuli are referred to as negative samples. TP indicates the number of positive samples correctly predicted. FN indicates the number of positive samples incorrectly predicted as negative samples. FP indicates the number of negative samples incorrectly predicted as positive samples. TN indicates the number of negative samples correctly predicted. For the convenience of observation, the confusion matrices reflecting the overall performance of LDA and SVM are given in Figure 8 after normalization.

**Table 2:** Confusion matrix for a two-class problem.

	**Prediction**	
**True**	**Target**	**No-target**
Target	TP	FN
No-target	FP	TN

We used another four exact criteria derived from the confusion matrix, sensitivity, precision, specificity, and F-measure, to evaluate the performance of the method as follows:

(12) $$\begin{array}{rcl} Sensitivity & = & \;\frac{TP}{TP+FN} \end{array}$$

(13) $$\begin{array}{rcl} Precision & = & \frac{TP}{TP+FP} \end{array}$$

(14) $$\begin{array}{rcl} Specificity & = & \;\frac{TN}{TN+FP} \end{array}$$

(15) $$\begin{array}{rcl} F_{measure} & = & \;\frac{2\times Precision\times Sensitivity}{Precision+Sensitivity} \end{array}$$

Sensitivity and specificity mean the ability to predict positive samples and negative samples, separately. Precision refers to the proportion of samples predicted positive are predicted correctly. Sometimes Sensitivity and Precision will contradict, so we choose F-measure that combines both of them. The results of LDA and SVM for each participant are shown in Tables 3, 4, respectively. From the results, the sensitivity of SVM is significantly higher than LDA (*p* < 0.01). The difference of precision is small (*p* > 0.05) but the F-measure of SVM is higher obviously (*p* < 0.05). Both specificities of them are very high.

**Table 3:** Sensitivity, precision, specificity, and F-measure of LDA for different participants.

**Participants**	**Sensitivity**	**Precision**	**Specificity**	**F-measure**
P1	0.5170	0.9282	0.9920	0.6641
P2	0.6560	0.9563	0.9940	0.7782
P3	0.5140	0.9362	0.9930	0.6637
P4	0.4940	0.9165	0.9910	0.6420
P5	0.4780	0.8968	0.9890	0.6236
P6	0.6560	0.9704	0.9960	0.7828
P7	0.6250	0.9843	0.9980	0.7645
P8	0.5690	0.9192	0.9900	0.7029
Average	0.5636 ± 0.073	0.9385 ± 0.030	0.9929 ± 0.003	0.7027 ± 0.064

**Table 4:** Sensitivity, precision, specificity, and F-measure of SVM for different participants.

**Participants**	**Sensitivity**	**Precision**	**Specificity**	**F-measure**
P1	0.6440	0.9083	0.9870	0.7537
P2	0.8190	0.9010	0.9820	0.8580
P3	0.7030	0.9336	0.9900	0.8021
P4	0.6360	0.9550	0.9940	0.7635
P5	0.8000	0.9816	0.9970	0.8815
P6	0.8030	0.9305	0.9880	0.8621
P7	0.7170	0.9111	0.9860	0.8025
P8	0.6830	0.8723	0.9800	0.7661
Average	0.7256 ± 0.073	0.9242 ± 0.034	0.9880 ± 0.006	0.8112 ± 0.050

*The sensitivity (p < 0.01) and F-measure (p < 0.05) are significantly higher than LDA. The difference between their precisions is small (p > 0.05)*.

**Figure 9:**
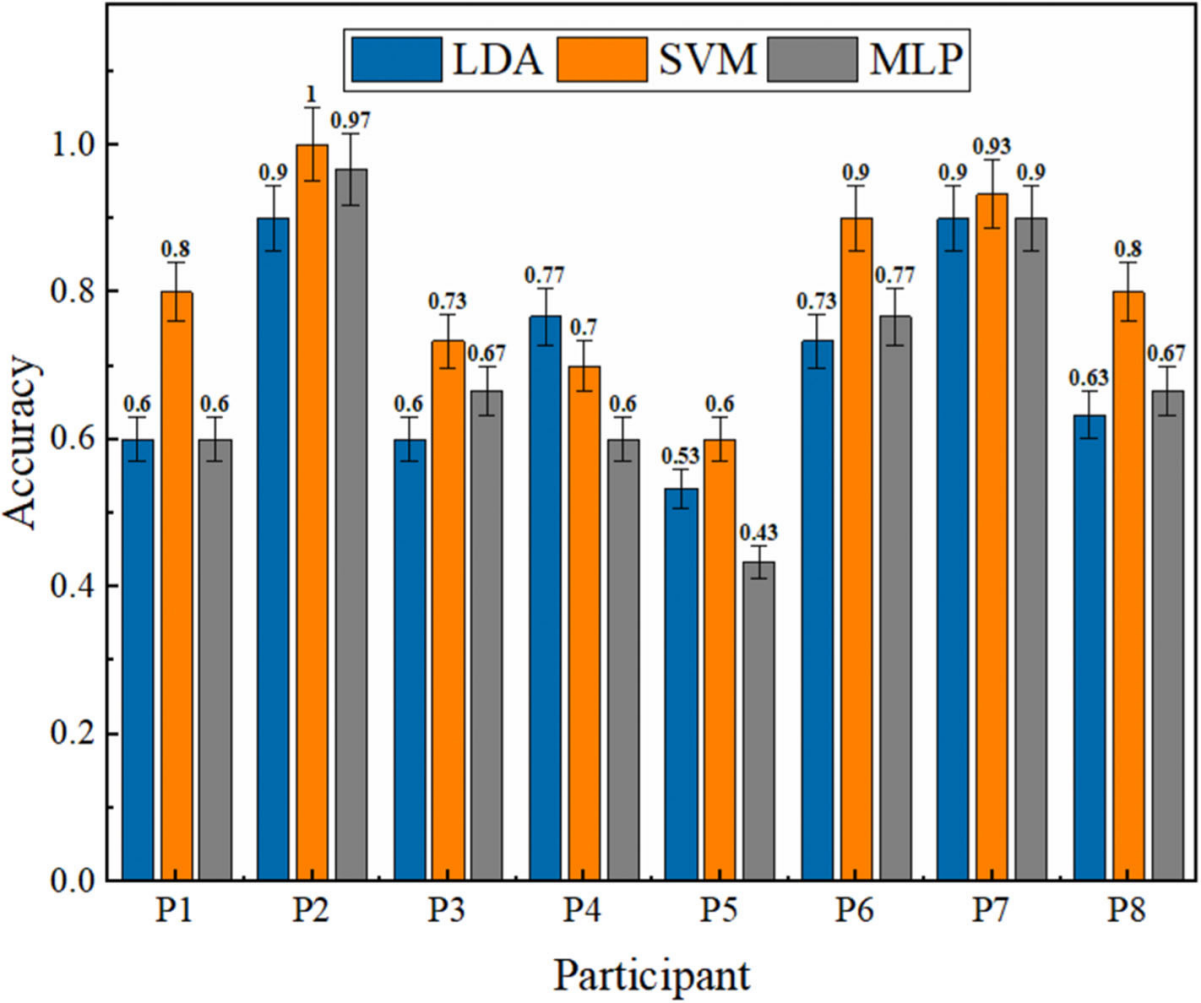
Online results of the three methods in eight different participants. The average accuracies with standard deviations of LDA, SVM, and MLP are 70.83±14.00, 80.83±13.18, and 70.00±17.28%.

The online tests with 30 characters in three groups per classifier were also conducted for each participant to test the actual performance of the method. As shown in Figure 9, the accuracies of the three classifiers are 70.83±14.00, 80.83±13.18, and 70.00±17.28%. The online results are lower and more unstable than offline, possibly due to different external factors and brain changes during thinking. The trends are similar to the offline test, in which SVM shows distinct superiority and robustness than LDA and SVM.

## Discussion

This paper aims to design a practical HMI to help disabled people control assist robot for some daily movements or rehabilitation exercises on their own. An EEG-based method is proposed with an effective spatial filter followed by a robust P300 classifier to obtain the user's intention and control the robot.

Since the characteristics of the EEG signal are still not well-understood, three representative classifiers, including LDA and SVM, that have distinct linear and non-linear features, as well as the MLP with neural network properties were chosen. The parameters of the classifiers determined by pre-experiment were consistent across all experiments to make certain the universality of the method. According to offline and online results, both LDA and SVM remained stable, but MLP varied wildly across different participants. Although the neural network has achieved many excellent classifications, it depends heavily on data size and hyperparameter fine-tuning. If the structure is simple, this method may not be able to handle all the features; however, if the structure is too complicated, it may consume too much time and also have the individual specificity problem, which is unacceptable in this control scenario. In further reference to the metrics derived from the confusion matrix, the overall performance of SVM is indeed preferable.

Our finalized method has achieved an offline accuracy of 94.43% and an online accuracy of 80.83%. Besides, the extended test of the mobile robot reached an online accuracy of 81.67%. The reliability and stability of our method make it more likely to be used in practice. Another valuable achievement is that the specificity is very high. This means that our control system hardly recognizes unexpected intention when the user does not need it, which may cause sudden movement of the assist robot. This character greatly guarantees the safety of users.

For the practical application of EEG for rehabilitation, time consumption is a problem that cannot be ignored. As shown in Table 5, the experiment takes only approximately half an hour for preparation, which is very convenient for the users, especially for people with mobility impairment and their escorts. Moreover, the ITR introduced by Shannon (1948) is used to quantitatively evaluate the efficiency and speediness. This metric, commonly used for measuring control systems, is defined as:

(16) $$\begin{array}{r} ITR=\frac{60\;\left(P\log_{2}P+\left(1-P\right)\log_{2}\left(\frac{1-P}{N-1}\right)+\log_{2}N\right)}{T}\left(bits/\min\right) \end{array}$$

where P is the probability of correctly recognizing a command (character), N is the number of classes, and T is the time needed to detect a command. Table 6 shows a comparison between previous research using the P300 signal and our method. Here, the N is 36, which supported our method to meet the practical needs of versatility. However, in this case, how to balance the accuracy and detection time becomes a difficult problem. To ensure stability and safety, ISI is set to 150 ms during training to help the participant adapt easier and is set to 75 ms during the online testing and actual use for faster speed. In comparison, we can see that although the offline performance is average due to the conservative T setting, the online performance has achieved a significant advantage. The method proposed by Cecotti et al. uses a massive data set to train a convolutional neural network with great potential (Cecotti and Graser, 2011). But as seen in this paper, there may be serious instability in online use.

**Table 5:** Time for preparation and detection.

**Preparation**	**Time (min)**
Lower impedance	25 min
Collect training data	8 min
Train model	1 min

**Table 6:** Comparison of offline and online ITR between our method and others.

**Reference**	**Year**	**Type**	**ITR**
Cecotti and Graser (2011)	2011	Off	8.25
		On	—
Akram et al. (2015)	2015	Off	7.03
		On	—
Han-Pang (2015)	2015	Off	—
		On	7.91
Kobayashi and Sato (2017)	2017	Off	8.67
v		On	—
Chen et al. (2017)	2017	Off	13.95
		On	—
Nurseitov et al. (2017)	2017	Off	16.53
		On	5.84
Achanccaray et al. (2019)	2019	Off	6.49
		On	4.54
Mao et al. (2019)	2019	Off	10.53
		On	—
Our method	2019	Off	10.13
		**On**	**15.42**

*Bold values highlights the best result of this paper*.

Our method has been significantly improved compared with some nice research based on P300, which was not limited to the use of assist robot. It can be seen that it achieves not only a high-performance method but also a practical and easy-to-use system for target groups from the experimental test, statistical inference, and comparison with other methods.

There are still some limitations. Some parameters in the method were not detailed for demonstration because we chose the best of the ones we had considered through a series of pre-experiments for comprehensive performance. And the number of repetitions used to detect a character was set to medium, that also made the T slightly long, to ensure our method was robust enough for any user. Besides, the inherent characteristics of P300 made the ITR less than some methods based on SSVEP. Nevertheless, SSVEP was not very acceptable for some applicable scenarios, such that some cases do not need to be too fast but need to be more stable and comfortable to use. A shortcoming cannot be ignored is that we did not compare our method in-depth with some recent great P300 detection methods based on deep learning (Ditthapron et al., 2019). However, we think our methods on the basis of classical machine learning also have decent performance and are convenient to be implemented with lower computation cost, which is easier to be used in practice for relevant developers. We will try to use deep learning to improve our online control system in future work.

## Conclusions

In this paper, a remarkable EEG-based human-machine interface is proposed to online control assist robots for disabled people. We have accomplished a high-performance method using the P300 component to detect the user's intentions for control. This method with good accuracy and ITR proved to be effective and practical enough for real life by offline and online tests. Moreover, based on the control method, an upper-limb assist robot is developed to assist users to perform some activities such as grasping, book turning with security measures and a user-friendly interactive program, which gives a meaningful reference to future work. Besides, a novel mobile robot controlled by our method and computer vision proves the robustness and generalizability. Further tests on stroke patients performing therapeutic exercises will be considered with the upper-limb assist robot.

## Data Availability Statement

The datasets generated for this study are available on request to the corresponding author.

## Ethics Statement

This study was carried out in accordance with the recommendations of SCUT Research Ethics Guidelines and Researcher's Handbook, Ethic Board of Medical school, South China University of Technology with written informed consent from all subjects. All subjects gave written informed consent in accordance with the Declaration of Helsinki. The protocol was reviewed and approved by the Ethic Board of Medical school, South China University of Technology.

## Author Contributions

YS, SC, and LX contributed conception and design of the study. YS, LY, and GL carried out the experiments, data processing, and assist robot design. YS wrote the first draft of the manuscript. SC, LY, GL, WW, and LX wrote sections of the manuscript. All authors contributed to manuscript revision, read, and approved the submitted version.

## Conflict of Interest

The authors declare that the research was conducted in the absence of any commercial or financial relationships that could be construed as a potential conflict of interest.
